# Effects of model-mimic frequency on insect visitation and plant reproduction in a self-mimicry pollination system

**DOI:** 10.1093/aobpla/plx044

**Published:** 2017-10-11

**Authors:** Rubem Samuel de Avila, Suiane Santos Oleques, Brisa Marciniak, José Ricardo I Ribeiro

**Affiliations:** Universidade Federal do Pampa, campus São Gabriel, Laboratório de Estudos em Biodiversidade Pampiana (LEBIP), Avenida Antônio Trilha, 1847, São Gabriel, Rio Grande do Sul, Brazil; Universidade Federal do Rio Grande do Sul, Programa de Pós-Graduação em Botânica, Laboratório de Taxonomia (Labtax), Brazil

**Keywords:** Fruit set, honeybee, Pampa biome, pollination, self-mimicry, sex ratio

## Abstract

The proportion of mimics and models is a key parameter in mimetic systems. In monoecious plants with self-mimicry pollination systems, the mimic-model ratio is determined by the floral sex ratio. While an equal sex ratio (1:1) could provide the perfect balance between pollen donors and stigma surfaces able to receive the pollen, an unequal ratio could increase pollination by production of a greater number of rewarding, model flowers. The aim of the present study is to test the differences in visitation frequency and reproductive rates of different mimic and model flower arrays in order to assess the efficacy of the mimetic system in a *Begonia cucullata* population. The frequencies of visitors to groups of flowers with three distinctive sex ratio arrays (male-biased, female-biased and equal ratio) were compared using a Bayesian approach. The reproductive outcomes were compared in order to detect advantages of particular sex ratios. Low visitation frequency was recorded in all arrays. Pollinators showed similar behaviour regardless of sex ratio; they tended to avoid female, rewardless flowers. Pollination quality was highest in the equal sex ratio array. The current study shows that sex ratio plays a critical role in the pollination of *B. cucullata* and that the efficacy of the self-mimicry system appears to be doubtful. Visitation frequency may be associated with visual or chemical cues that allow pollinators to recognize models and mimics, regardless of their frequency in the population.

## Teaser

Offering floral rewards to pollinators such as nectar and pollen may not mean reproductive success. Some plant species present strategies to minimize this cost and dupe their pollinators. Begonia cucullata presents a mimicry system characterized by female rewardless flowers (mimic) that mimic male flowers (model) with a large amount of pollen. For the perfect functioning of mimetism, the proportion of mimics should not be higher than models. We tested different arrays of mimic-model flower proportions to test the efficacy of this system. We observed that bees avoided rewardless female flowers, although fruit set did not indicate pollen limitation.

## Introduction

Different strategies have evolved for the optimization of reproductive rates in flowering plants. These range from vegetative and clonal growth to extremely specialized interactions with pollinators. The interactions with animal pollinators generate advantages in different ecological contexts, but they involve trade-offs that include several types of resources available to animal vectors. There is thus the need to estimate both production costs and the losses resulting from inefficient pollen vectors and predation for inclusion in models of selection generating pollination strategies (Obeso 2002).

An interesting pollination mode that is able to partially minimize the production costs of floral rewards is found in plant species using rewardless flowers to deceive pollen vectors. These mimetic pollination systems are widespread among angiosperms (≈32 families) (Baker 1976; Renner 2006). One of these systems is the self-mimicry pollination system (or sexual mimetism) (Dafni 1984), which is a type of floral mimetism with fundamental features (Williamson 1982): a floral morph model that presents rewards (model) and a floral morph without rewards to mimic the model and deceive the floral visitors (mimetic). The animal should not be able to recognize the mimetic flowers for this pollination mode to be effective; such effectiveness is assured by the flowers’ chemical and morphological traits (Williamson 1982; Barrett 1987; Schaefer and Ruxton 2009).

Unlike animal mimetic systems, in plants the proportion of models and mimics in self-mimetic systems is associated with the sex ratio. The sex ratio of models (male flowers) to mimetics (female flowers) depends on a mating system, which assures seed set and minimizes plant population inbreeding (Bullock and Bawa 1981; Agren *et al.* 1986). Sex ratios can vary widely within plant populations depending on different resource conditions or on the quality of pollination, for example (López and Domínguez 2003; Barrett *et al.* 2010). However, variations in the sex ratio in the mimetic pollination mode can make the system more vulnerable to pollinators that recognize the rewardless floral morphs, and can decrease the efficacy of mimetism.

*Begonia cucullata* is a monoecious species presenting pollen-bearing male flowers and rewardless female flowers in the same individual. Studies on the floral biology, reproductive traits and effective pollinators have been conducted (Wyatt and Sazima 2011); however, there is insufficient information on the frequency of the two morphs and their reproductive effects on this species. Castillo *et al.* (2002) investigated these aspects and found density and frequency dependence on the visitation to and reproductive rates of *Begonia gracilis.* They also found that selection dependent on density and frequency acted simultaneously to determine the expected floral morph ratio in this intersexual mimetic system. López and Domínguez (2003) also showed that the sex ratio could be determined by reproductive outputs in *B. gracilis.*

Although similar experiments to those conducted here have already been published (Castillo *et al.* 2002), none of them adopted Bayesian methods for the data analysis. Conventional data analysis approaches are known as frequentist statistics, and include null hypothesis significance testing (NHST) and the generation of confidence intervals (McCarthy 2007; Kruschke 2010a). The use of a Bayesian approximation in the female-flower visiting model is a promising approach, because relevant information can be naturally incorporated into such Bayesian analyses through the specification of appropriate probabilities for the relevant parameters (McCarthy 2007). Bayesian methods provide tremendous flexibility for data analytic models and generate rich information about parameters that can be used cumulatively in subsequent experiments (Kruschke 2010a). The statistical inference about the parameter of interest is presented as changes in the uncertainty about its value in light of evidence. The Bayes theorem specifies how this change should take place (Kinas and Andrade 2010); therefore, the parameters are treated as random variables within the Bayesian paradigm—a description of the uncertainty about these parameters’ true values is provided accordingly (Bernardo 2003). Finally, large samples are not actually necessary for a Bayesian data analysis, although they are often useful as approximations and instruments to help understand the analysis.

On the other hand, the *P*-values in frequentist approaches do not necessarily provide reliable indicators to support the null hypothesis, because frequentist methods overall and the *P*-values in particular do not provide direct statements about the reliability of the hypothesis (Berger and Berry 1988). Instead, they provide direct information about frequency of data occurrence, and thus only indirect support or otherwise for the hypothesis (McCarthy 2007). Finally, a crucial problem concerning the use of NHST may lie in the fact that a single set of data could have been derived from many different experiments; therefore, a single set of data could present many different *P*-values (Kruschke 2010a).

In the light of the assumptions proposed by Castillo *et al.* (2002), the aim of the present study was to investigate whether sex ratio variations (male flowers, i.e. models, and female flowers, i.e. mimics) affect the frequency of visitors and reproductive success in a *B. cucullata* population growing in Southern Brazil, based on a hierarchical Bayesian framework. Moreover, it aimed to assess whether the dupe is a fundamental requirement for mimetic systems to operate efficiently. The following predictions were made: visitor frequency to the male (model) and female flowers (mimic) of an even sex ratio population would be equal; more dupes would be found in male-biased (models) populations, leading to a lower risk of pollinator discrimination towards female flowers and representing reproductive gains for the *B. cucullata* population; the opposite effects would be expected in a female-biased population with a lower number of dupes, a higher risk of female rewardless flower discrimination and a corresponding decrease in reproductive rate. Since different insects may visit different flowers, the goal was to assess whether the three treatments presented different within-group variations through the adoption of different assumptions based on the Bayesian hierarchical framework and on the comparison of such models.

## Methods

### Studied species

The present study was conducted in an area of São Gabriel County (30°20′41.23″ S and 54°20′10.53″), Rio Grande do Sul, State - Brazil, in November 2014. The county belongs to the country’s Southern region, which is characterized by a sub-temperate climate and by the undulating grassland typical of the Pampa biome.

Most species belonging to the genus *Begonia* (Begoniaceae) present staminate and pistillate flowers within different anthesis phases (Agren and Schemske 1993); the staminate flowers often open before the pistillate flowers (Wyatt and Sazima 2011). Female flowers do not present rewards and mimic the male flowers. The tepals and stigma of female flowers typically resemble the tepals and the androecium of male flowers, both in size and colour (Schemske and Agren 1995; Schemske *et al.* 1996).

*Begonia cucullata* is a monoecious herb of broad distribution in Argentina, Paraguay and Brazil (except in the Northern region). It inhabits disturbed wet areas such as roadsides and riverbanks (Kollmann 2006) and presents cymose inflorescences composed of male and female flowers with colours ranging from white to light pink and red. A continuous flowering pattern and long flowering peak of ~6 months have been described in other studies (Wyatt and Sazima 2011).

### Differential sex ratio: experimental design

Three treatments, with 10 individual plant patches each, and ~10 ramets in each patch, were applied to a natural *B. cucullata* population. The patches exhibited similar plant density and were spatially separated in order to minimize spatial autocorrelation and to increase data independence. The ‘control treatment’ (CT) was a population with an even sex ratio (1:1). The other two treatments had an uneven sex ratio. The sex ratio in the patches of the ‘more-female-flowers treatment’ (MFT) was hand-manipulated to reduce the number of male flowers to the ratio 1:3; likewise, the number of female flowers in the ‘more-male-flowers treatment’ (MMT) was reduced to give a male:female ratio of 3:1. A total of 100 h of field observation were conducted; each flower visitor was observed within patches and the sequence of visits to male and female flowers was recorded as a trail.

### Reproductive tests

One hundred flowers from 10 individuals in each treatment (i.e. CT, MFT and MMT) were marked for assessment of natural reproductive success (fruit set = fruits formed/marked flowers). The marked flowers were observed until the unripe fruiting stage. Pollen limitation (PL) was tested in the CT plants and, in order to do so, 19 buds from five different plants were isolated in voile bags and hand-pollinated with pollen from a different individual. Pollen limitation was defined as a significant difference between the fruit set produced by natural pollination in the CT and that produced by hand pollination (*t*-test, *P* < 0.05). Twenty buds from each of the three treatments were closed and hand-pollinated to determine the occurrence of self-pollination. The fruits from each treatment (i.e. CT, MFT, MMT, hand cross-pollination and hand self-pollination) were weighed to assess fruit quality.

### Visitor behaviour and statistical analyses

#### The frequentist approach.

The fruit set and fruit dry weight values in each treatment were compared through one-way ANOVA and Tukey’s test. The *t*-test was used to compare the fruit set resulting from hand pollination and natural pollination in order to assess PL.

#### The Bayesian approach.

The probability of the insect not recognizing the mimetic flowers (without resources), given the number of female flowers visited, was inferred through estimated unknown parameters. These parameters were the possible distributions, denoted as ‘posteriors’ (Gelman *et al.* 2003), within a hierarchical modelling approach (Kruschke 2010a) based on the Bayesian inference. Accordingly, a hierarchical Bayesian framework was used to accommodate the uncertainties of the herein proposed hyper-parameters of the models (i.e. the distribution parameters were assigned as hyperpriors, which were, in turn, random variables).

Each visitation sequence (a trail) corresponded to a ‘coin’ flipped *n* times, as long as each of the sequences was being followed. Accordingly, the number of correct choices (i.e. female flower being visited) of insect *j* from experiment *e* was binomially distributed, because the data were the sums across trails rather than individual trails presenting parameter *θ*, which represented the probability of the insect being duped by the female flower. Instead of having a single *θ* value, the bias of the *j*th insect from the *e*th experiment was denoted *θ*_*je*_. The total number of visits to female flowers was denoted *z*_*je*_ (i.e. the number of correct choices by the *j*th insect from the *e*th experiment), because the individual visits were performed by different insects from different experiments.

The insects were assumed to randomly represent the group of all insects, and their respective trails. The group within a treatment had a mean ability to distinguish female from male flowers, and such ability was denoted by the hyper-parameter *μ*; the typical accuracy of the group was based on its distribution propensities to visit the female flowers. The dependence of individual abilities on the mean group of experiments *μ* was measured through the hyper-parameter *k* (i.e. the link between individual propensities and the group’s mean). Therefore, if the insects’ choices were identical, then the lack of variation between insects would suggest that the visiting female flower bias *θ* was strongly dependent on the ability bias *μ*; hence, the posterior mean *k* value would be higher than its adopted prior mean (Kruschke 2011). In other words, the lack of variation between insects suggests that the insect biases *θ*_*j*_ are strongly dependent on the discriminatory ability bias *μ*. Different insects could have different individual reactions induced by their discriminatory ability, but the reaction may be dependent on the overall effect of how such a mimic system acts in these insects, and on how these insects are affected.

The *θ*_*je*_ values, therefore, depended on the value of the hyper-parameters *μ* and *k* of the visiting insects’ biological features. A β distribution defined by the shape parameters *A*_*μ*_ and *B*_*μ*_ was used to establish the prior for the *μ* parameter; whereas the *k* parameter had a prior that has a γ distribution defined by the shape and rate parameters *S*_*k*_ and *R*_*k*_ according to the hierarchical dependence model described by Kruschke (2011) (see details below). Thus, the Bayesian inference about mimetism efficacy, given the number of female flowers visited, is mainly focused on estimating the joint conditional posterior probability distribution of *μ* and *k*. Therefore, the herein adopted Bayes rule was *p*(*θ*, *μ*, *k*, *θ*_*μ*_, *θ*_*k*_ | *z*); wherein the *θ*_*μ*_ and *θ*_*k*_ parameters were the priors in the ability bias of distinguishing female from male flowers, as well as in how strongly *θ* depends on *μ*. Thus, at least the *J* + 2, *θ*_1_,…,*θ*_*j*_, *μ* and *k* parameters of each experiment were simultaneously estimated, as well as the *μ* shape parameters of the β distribution (its prior distribution), and the *k* shape and rate parameters of the γ distribution (its prior distribution).

The posteriors were found through the likelihood of data integration with other relevant information expressed in prior probability distributions by means of the Bayes’ theorem. The posteriors were approximated using random samples taken from the integration of the data likelihood with the prior probability distributions, since analytical solutions were not feasible. The inclusion of external information to establishing the prior was not the unique possibility. Appropriate non-informative or ‘open-minded’ priors for the parameters were also herein adopted, but they were limited to the positive range values. Accordingly, on the assumption that distinguishing female from male flowers is relatively uncommon, since it occurs in other species, a β distribution was used based on the parameters *a* = 4 and *b* = 1, herein called ‘Condition 1’ (C1) with the shape parameters *A*_*μ*_ and *B*_*μ*_. Due to the expectations about insects tending to be deceived by female *B. cucullata* flowers (see details about intraspecific Bayesian mimicry in Dafni (1984)), *m* = 0.80 was considered the mean Bayesian mimicry success value. Thus, the prior in *μ* has 0.80 expectation, and *n* = 5 is the number of visits required to confirm the outcome; so, *mn* = *a* = 4 and *b* = (1 − *m*)*n*, *b* = 1, according to the procedure suggested by Kruschke (2011). Non-informative priors (‘open-minded’ priors) were used, and their use was possible through the adoption of a uniform distribution (or a β distribution using parameters *a* = 1 and *b* = 1), which was herein called ‘Condition 2’ (C2).

Different assumptions concerning *k*_*e*_ in the present analyses were adopted, according to Kruschke’s (2011) protocol, in order to assess the effects of different across-group constraint assumptions. Firstly, the overarching distribution variance was estimated based on the data; therefore, if the data in most groups were close to each other, the estimate of the overarching distribution variance estimate would be low. Such an approach may minimize false alarms by shrinking the outlying condition estimates and forcing them towards the grand mean (Kruschke 2010b, 2013). Thus, if individuals’ accuracy in each experiment depends on the group’s mean accuracy, the magnitude of individuals’ dependence on the group’s mean would be estimated. However, one may assume that whatever the degree of dependence is, it tends to be similar between groups, and the data inform such estimate similarity.

Accordingly, the present study addressed whether or not the different insect individuals observed in each experiment were equally affected by the flowers, since individuals in all experiments were treated as independent samples from a common group of insects presenting similar discriminatory abilities. The shrinkage magnitude was represented by the data; such magnitude was herein measured as the standard deviation (*σ*_*k*_) of *k*. Therefore, the magnitude was expected to be lower than or equal to the SD suggested in the present study, namely: *σ*_*k*_ = 10.0 (see model A2 below). This SD was considered to be the hierarchical model parameter, and its structure was separately copied in each condition.

Secondly, a model wherein the same *k* value was simultaneously used in all groups was adopted as an alternative. Therefore, if individuals’ accuracy in each group depended on the group’s mean accuracy, the magnitude of such dependence on the group’s mean was estimated. However, regardless of the degree of dependence, the magnitude was the same in every group. Accordingly, this assumption is the same as saying that the category structure (i.e. CT, MFT and MMT) affects the mean accuracy of the group. On the other hand, individual variations based on the mean accuracy are caused by other factors, rather than by the category structure. It is worth emphasizing that these factors are the same across groups; in other words, differences from insect to insect are allowed, but these differences were not expected to have predictable effects favouring one insect in detriment to another, *a priori*.

Finally, a model considering each category structure was adopted. The structures have their own mean discriminatory ability, as well as their own scores of individual spreading around the mean; in other words, data from a single treatment had no influence on data from another treatment.

Hence, model A1 was denoted as a hierarchical model that constrains the hyper-parameter *k* of every condition to the same value. Model A2 is a hierarchical model and its structure was separately copied in each condition. Both models were herein classified as naïve models. Model B is a hierarchical model; its values were mutually informed by overarching distribution estimates. The shape and rate of γ distribution parameters in models A2 and B were re-expressed as the mean *μ*_*γ*_ and the standard deviation *σ*_*γ*_. The mean and SD, in turn, were given uniform prior distributions over a positive interval in model B. Therefore, if data indicate that the individual variation within each group is almost identical across experiments, then the estimated *k*_e_ values should be narrow, so that such narrowness, in this case, would be captured by the estimated *σ*_*γ*_, and would be lower than 10.0. The prior in *k* had a mean, *μ*_*γ*_, of 10.0, and a standard deviation, *σ*_*γ*_, of 10.0 in model A2, thus, allowing great variation between insects’ discriminatory abilities due to the low *k* values. If one considers that the posterior is a joint probability distribution that specifies the credible combinations of all parameter values in a hierarchical model, it is possible to address the following question: how big is the difference between experiments when the credible combinations of *μ* hyper-parameter values are compared to each other?

Posterior distribution samples were drawn according to the Markov chain Monte Carlo (MCMC) method (Gelman *et al.* 2003). A Markov chain in MCMC is determined in such a fashion that the posterior is its long-run equilibrium distribution. The posterior’s means were used as parameter estimates, unless otherwise stated. Four (4) chains with 50000 iterations each were run, the first 10000 iterations of each chain were discarded as burn-in, except for model B. One hundred thousand (100000) iterations of each chain were adopted in model B and 40000 iterations of it were discarded. The visual inspection means and the Gelman and Rubin (1992) protocols were used after confirming that the chains had converged to collapse the samples across the four chains. The uncertainty of these estimates was expressed through 95 % posterior probability intervals, with lower and upper limits equal to quantile 2.5, and with 97.5 % of the posterior sample, respectively (i.e. 95 % of the highest density interval (HDI) by Kruschke (2011)). Each histogram was annotated with its central tendency (a mean for roughly symmetrical distributions and a mode for skewed distributions if this was the case). The posterior probability interval is the Bayesian analogue to the conventional confidence intervals (Ellison 2004; McCarthy 2007); every value inside the HDI has a higher probability density than any value outside it (Kruschke 2013). The model selection was based on the deviance information criterion (DIC) (Spiegelhalter *et al.* 2002). All Bayesian analyses were performed in the rjags and runjags packages, which were implemented by the R Development Core Team (2015) and by JAGS (Just Another Gibbs Sampler) (Plummer 2003).

## Results

### Floral visitors and sex ratio

**Figure 1.**
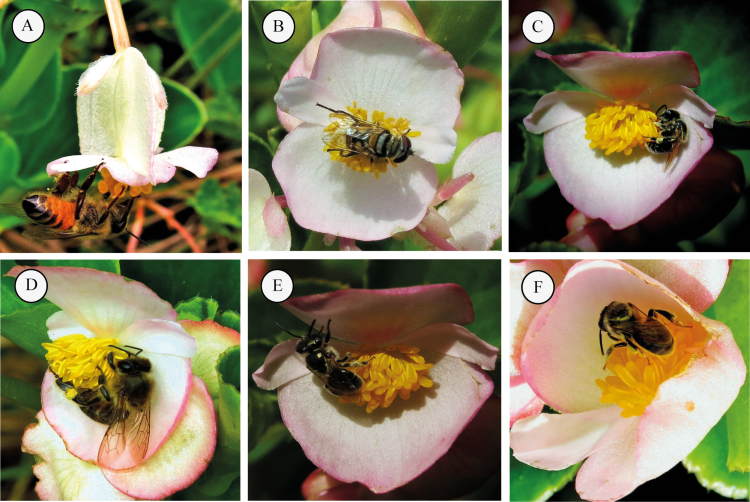
Floral visitors observed in *Begonia cucullata* flowers: (A) *Apis mellifera*, in female flower. (B) Fly *Palpada* sp. (Syrphidae). (C) *Plebeia* sp. 1. (D) *Apis mellifera*, in male flower. (E) *Plebeia* sp. 2. (F) *Plebeia* sp. 3.

The sex ratio (male:female) observed in the studied natural population was ~1:1 (1.17:1); chi-square test χ^2^ = 10.151, df = 9, *P* = 0.338. A total of 813 floral visits (442 in CT, 189 in MMT and 182 in MFT) and 118 trails (51 in CT, 36 in MMT and 31 in MFT) were observed **[see Supporting Information—Table S1]**. Honeybees (*Apis mellifera*), Meliponinae bees (*Plebeia* spp.), flies (Syrphidae, *Palpada rufipedes*) and beetles (Chrysomeliidae, *Diabrotica speciosa*) were recorded visiting *B. cucullata* flowers at the study site (Fig. 1). Honeybees were the most numerous visitors to *B. cucullata* flowers in the non-manipulated population (CT) (84.61 %) and in the other two treatments (94.87 % in MMT and 81.25 % in MFT).

### Sex ratio: does this affect female flower visits?

Insects systematically selected male instead of female flowers, apparently regardless of the sex ratio. The mean ability values, denoted by 1 − *μ*, were 0.89, 0.83 and 0.93 in treatments CT, MFT and MMT, respectively (Table 2).

Based on DIC, the hierarchical naïve model A2-C2, presenting open-minded priors [i.e. *μ* ~ *β*(*μ* | 1, 1)], was the one best fitting the data (Tables 1 and 2). In this model, one experiment did not constrain the others, so that *k*_1_, for example, was free to be higher than *k*_2_ or *k*_3_, whereas *k*_2_ was randomly selected to be lower than *k*_1_ or *k*_3_. The posterior in the *μ* found through this model indicated that a high chance of the aforementioned mimicry being found in the system (i.e. ~80 %, see above) was not in the 95 % most believable values (Table 2). Therefore, the posterior indicates that the group’s most believable accuracies actually tend to have less chance of being correct. Additionally, the wide uniform prior in *μ* became the already mentioned smoothly peaked and relatively narrow posterior distributions. This means that the posterior *κ* means were higher than the mean prior in all experiments, thus indicating that insect accuracies were more similar to each other than the assumed prior (i.e. mean value higher than 10.0) (Table 2). However, the posterior *k*_2_ mean (i.e. treatment MMT) was not much higher than 10.0, unlike the other posterior *k* mean. The absence of correlation in the scatterplot of *k* by *μ*, and the lowest credible value of the *k* estimated (13.34 [2.24; 40.57]) in treatment MMT indicated that these insects can spread a little further from the group’s mean than insects in the other experiments.

**Table 1.** Bayesian fits of the two models for mimicry success in male flowers of *Begonia cucullata* when prior has *μ* ~ *β*(*μ* | 4, 1). We considered *κ* ~ *γ*(*κ* | 1.0, 0.1) in the model A2. The symbols *μ*, *κ* and *σ*_*γ*_ represent the following estimated posterior hyper-parameters: *μ*, the mean ability in distinguishing female flowers from male flowers; *κ*, the dependency of the individual abilities on the group experiment mean, such that if the insect’s choices are identical, then such lack of variation between insects suggests that visiting female flowers’ biases, *θ*, are strongly dependent on the ability bias *μ*; *σ*_*γ*_, standard deviation of *k*, so that it is expected to be smaller or equal to that suggested here (indicated by an asterisk) as parameter of a hierarchical model with its structure copied separately for each condition. Values within brackets are the 95 % probability intervals (i.e. 95 % HDI), respectively. Bold value refers to the best model. DIC is the deviance information criteria; a smaller DIC indicates a better fit. CT (control treatment: ratio 1:1), MMT (more-male-flowers treatment: ratio 3:1), MFT (more-female-flowers treatment: ratio 1:3).

Parameter	Naïve models		Based on an overarching distribution
*β*(*θ* | 4, 1)	Model A1	Model A2	Model B
*μ*_1_ (CT)	0.12 (0.09–0.16)	0.12 (0.09–0.16)	0.12 (0.08–0.15)
*μ*_2_ (MMT)	0.09 (0.05–0.14)	0.10 (0.05–0.17)	0.09 (0.05–0.15)
*μ*_3_ (MFT)	0.18 (0.12–0.25)	0.19 (0.13–0.26)	0.18 (0.12–0.25)
*κ*_1_ (CT)	—	23.07 (7.36–52.88)	29.85 (10.59–77.44)
*κ*_2_ (MMT)	—	12.48 (2.29–38.41)	22.85 (4.22–58.60)
*κ*_3_ (MFT)	—	16.50 (4.01–44.02)	25.45 (6.84–64.78)
*κ*_common_	23.39 (8.55–51.47)	—	—
*σ*_*γ*_	—	10.0*	11.79 (0.50–28.27)
DIC	234.0	**228.7**	234.9

**Figure 2.**
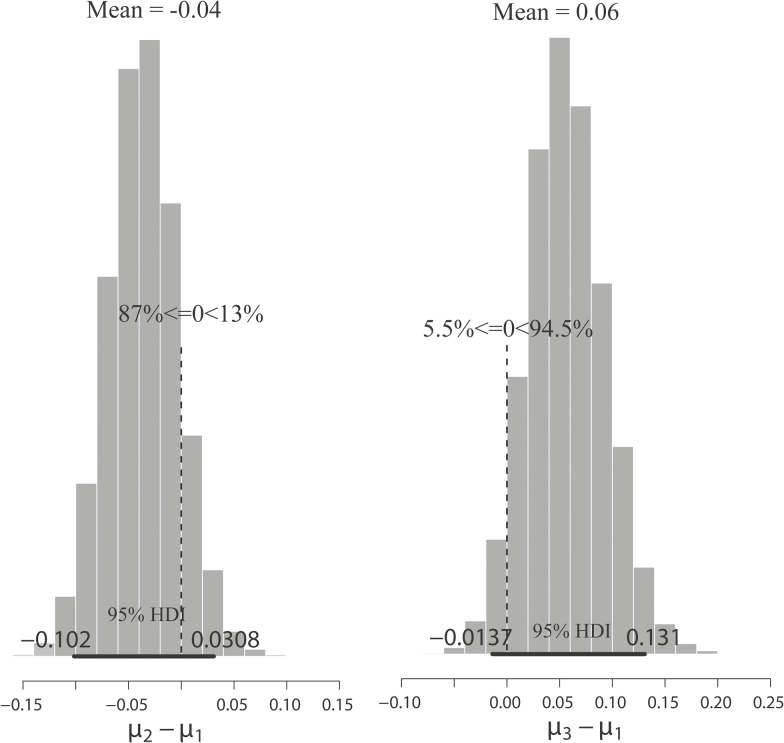
Histograms showing the posterior samples of different *μ* parameter values, from JAGS (Just Another Gibbs Sampler), between the different treatments: MMT (a male-biased population, *μ*_2_), CT (control treatment, *μ*_1_), MFT (a female-biased population, *μ*_3_) and CT. The difference ‘zero’ in the 95 % most credible values. Only 13.0 % of the credible values are >0 in the first histogram, whereas only 5.5 % of the credible values are <0 in the second histogram. 95 % HDI, 95 % highest density interval.

The histograms concerning the differences in the *μ* parameter values clearly show that the mean difference between the *μ*_2_ (i.e. the treatment MMT) and *μ*_1_ (i.e. the treatment CT) posteriors include zero in the 95 % most credible values (mean = −0.04 [−0.10; 0.03]), if one considers the hierarchical naïve model A2-C2 with open-minded priors (the best model). Therefore, it is possible that only 13 % of the credible values are higher than zero as well. The same is shown in the mean difference between the *μ*_3_ (i.e. the treatment MFT) and *μ*_1_ posteriors, although they are slightly higher than the first one (mean = 0.06 [−0.01; 0.13]). Although the difference in posteriors *μ*_3_ and *μ*_1_ indicates that the 95 % HDI does not exclude zero, ~95 % of the posterior is higher than zero (Fig. 2). The *k* variability and the absence of correlation in the scatterplot *k*_2_ by *μ*_2_, as well as the negative correlation scatterplot of other *μ*s by *k*s, produced greater variability in the *μ*s, and it led to weaker differences between *μ*s. Such correlation seems to be related to the individual accuracies in treatments CT and MFT, which are slightly skewed towards the big values; thus, if *k*_1_ or *k*_3_ are bigger, then the most credible *μ* is a little smaller **[see Supporting Information—Fig. S1]**.

The scatterplots of *μ* and *k* reveal that the *k* variability is slightly lower in the naïve model A1 than in the other models **[see Supporting Information—Fig. S1]**, and that it constrains *k* to be equal in all groups. The estimated overarching distribution (Table 2) has made the differences among *μ* apparently unchangeable in model A2-C2.

**Table 2.** Bayesian fits of the two models for mimicry success in male flowers of *Begonia cucullata* when prior has *μ* ~ *β*(*μ* | 1, 1). We considered *κ* ~ *γ*(*κ* | 1.0, 0.1) in the model A2. The symbols *μ*, *κ* and *σ*_*γ*_ represent the following estimated posterior hyper-parameters: *μ*, the mean ability in distinguishing female flowers from male flowers; *κ*, the dependency of the individual abilities on the group experiment mean, such that if the insect’s choices are identical, then such lack of variation between insects suggests that visiting female flowers’ biases, *θ*, are strongly dependent on the ability bias *μ*; *σ*_*γ*_, standard deviation of *k*, so that it is expected to be smaller or equal to that suggested here (indicated by an asterisk) as parameter of a hierarchical model with its structure copied separately for each condition. Values within brackets are the 95 % probability intervals (i.e. 95 % HDI), respectively. Bold value refers to the best model. DIC is the deviance information criteria; a smaller DIC indicates a better fit. CT (control treatment: ratio 1:1), MMT (more-male-flowers treatment: ratio 3:1), MFT (more-female-flowers treatment: ratio 1:3).

Parameter	Naïve models		Based on an overarching distribution
*β*(*θ* | 1, 1)	Model A1	Model A2	Model B
*μ*_1_ (CT)	0.11 (0.08–0.15)	0.11 (0.08–0.15)	0.11 (0.08–0.14)
*μ*_2_ (MMT)	0.07 (0.04–0.12)	0.07 (0.02–0.13)	0.07 (0.03–0.12)
*μ*_3_ (MFT)	0.16 (0.11–0.23)	0.17 (0.11–0.24)	0.16 (0.11–0.23)
*κ*_1_ (CT)	—	24.53 (8.06–54.95)	31.48 (11.74–81.46)
*κ*_2_ (MMT)	—	13.34 (2.24–40.57)	24.74 (4.59–65.00)
*κ*_3_ (MFT)	—	17.72 (4.37–46.12)	26.47 (7.74–68.29)
*κ*_common_	26.00 (9.69–56.96)	—	—
*σ*_*γ*_	—	10.00*	11.86 (0.55–28.35)
DIC	233.1	**227.3**	233.3

### Differences in sex ratio: are there effects on fruit set and dry weight of fruits?

**Figure 3.**
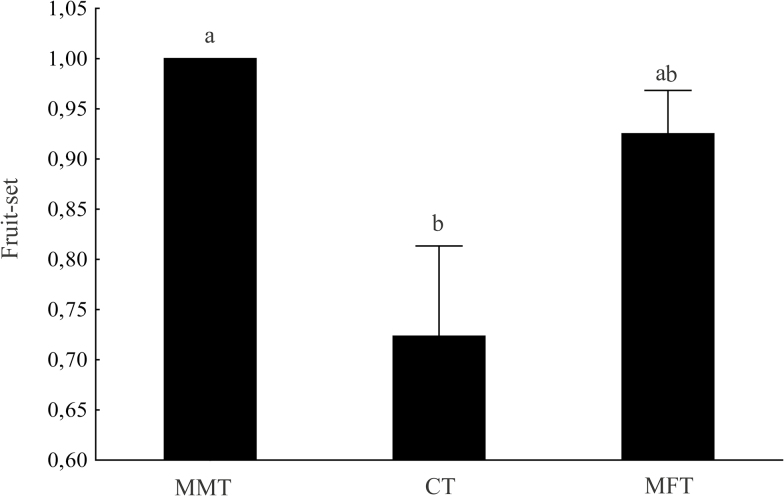
Mean (±1 SE) fruit-set values in the three different sex ratio treatments applied to the *Begonia cucullata* population. Different lower case letters on each column indicate a significant difference (0.05, Tukey’s test).

The highest fruit-set value was observed in MMT (fruit set = 1, all flowers set fruits), followed by MFT (fruit set = 0.92 ± 0.14) and CT (fruit set = 0.72 ± 0.28). There was a significant difference between MMT and CT (one-way ANOVA, *F*_2, 27_ = 6.165; *P* = 0.006) (Fig. 3); the *B. cucullata* population did not show PL according to fruit set (fruit set in hand pollination = 0.77 ± 0.40, and natural pollination = 0.72 ± 0.28; *t*-test, *t* = 0.310; df = 14, *P* = 0.7).

**Figure 4.**
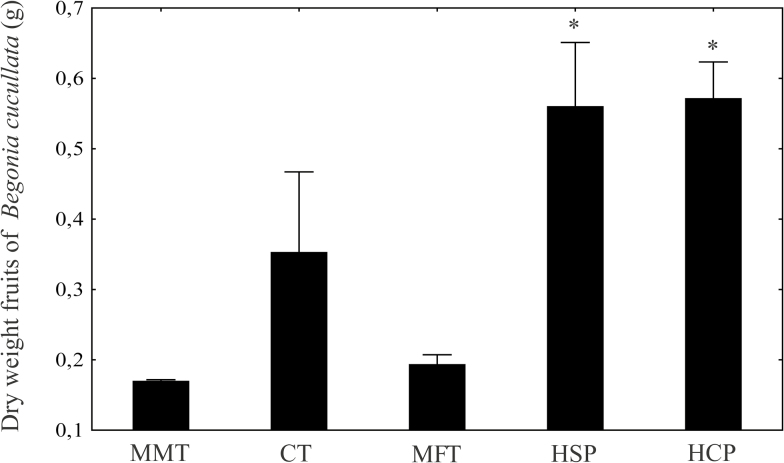
Mean (±1 SE) dry weight of *Begonia cucullata* fruits in the three different sex ratio treatments; self- and cross-pollinated manipulation. Significant differences (0.05, Tukey’s test) are indicated by an asterisk (*).

The three treatments showed no significant differences in fruit dry weight (MMT = 0.16 ± 0.007 g; CT = 0.35 ± 0.36 g; MFT = 0.19 ± 0.04 g; HSP = 0.55 ± 0.22 g and HCP = 0.57 ± 0.11 g; ANOVA, *F*_2, 27_ = 2.22; *P* = 0.13). However, the mean values of this parameter in these three treatments were lower than the HCP and HSP values of fruit dry weight (Fig. 4).

## Discussion

### Floral visitors

*Begonia* is a typically bee-pollinated genus, and bees are probably the main Begoniaceae pollinator because pollen is the main floral reward (Steiner 1976; Wiens 1978; Castillo *et al.* 2002; Wyatt and Sazima 2011). However, geographical differences may influence the pollinator assemblage in the same plant species. Wyatt and Sazima (2011) identified two bee species as *B. cucullata* pollinators in the Atlantic rainforest of Serra do Mar State Park (São Paulo, Brazil): *Augochloropsis* sp. and *Trigona spinipes.* Besides these two bee species, four other bee species (*A. mellifera*, *Plebeia* sp. 1, *Plebeia* sp. 2 and *Plebeia* sp. 3) were also recorded at the study site. *Apis mellifera* was the most frequent species, and possibly the most effective pollinator in all treatments; however, its efficiency has been questioned. Honeybees collect large amounts of pollen grain and exhibit extremely competitive behaviour, a fact that could be associated with the displacement of native bee populations (Aizen and Feinsinger 1994). The lack of PL as indicated by the fruit set found in the present study could be related to this, with the high frequency of honeybee visits assuring fruit set in the studied *B. cucullata* population. Other floral visitors such as Syrphidae and Coleoptera are common in *Begonia* flowers; although they effectively contact the stigmas and anthers, they can be considered secondary pollinators or pollen consumers that might remove the pollen deposited on the stigma during earlier visits (Schemske *et al.* 1996; Wyatt and Sazima 2011). The suppression of native vegetation by agricultural practices is notable at the study site; this could be associated with the high frequency of honeybee visits to *B. cucullata* flowers. Recent studies point out the higher resilience of honeybees facing anthropogenic disturbances associated with land use for agricultural purposes and for other economic practices compared to other native bee and insect species (Garibaldi *et al.* 2011). Although the fruit set did not indicate PL, the difference in mean dry weight values found between the treatments and the hand-pollinated groups suggest that PL is a real issue. Thus, the effectiveness of the pollinator service in this *B. cucullata* population is doubtful.

### Different sex ratios: is there an effect on visits to female flowers?

The extremely low probability of insects being duped by female flowers (mimics) is shown by the hyper-parameter *μ*. The group’s typical accuracy, based on the group’s distribution propensity to visit female flowers in all treatments (0.11 in the CT treatment, 0.17 in the MFT treatment and 0.07 in the MMT treatment), could be associated with the optimal foraging mechanisms able to decrease dupe costs. Such a pattern has been shown by many different plant species presenting rewardless flowers (Baker 1976; Agren *et al.* 1986; Dukas 1987; Le Corff *et al.* 1998). The herein cited studies showed morphological differences between sexes or phenological traits associated with the discrimination of rewardless flower pollinators. On the other hand, male and female *B. cucullata* flowers did not exhibit significant morphological differences (Wyatt and Sazima 2011). Furthermore, three distinctive phenological conditions in the sex ratio observed in this population were simulated, and the same low female flower frequency was found, conflicting with the present predictions. Castillo *et al.* (2002) found significant differences in visit frequency between female and male-biased sex ratios, thus differing from the current findings, and this could be associated with the main pollinators found in the two studies. Furthermore, simultaneous distractors, or other species in the community presenting floral rewards, may also operate in this system (Chittka and Raine 2006; Ma *et al.* 2015), and may have contributed to the frequency-dependence effects.

The mean difference *μ*_2_ − *μ*_1_ was slightly smaller than the mean difference *μ*_3_ − *μ*_1_, but both differences included zero in their 95 % most credible values (Fig. 2). Therefore, insects were systematically selecting male flowers rather than female flowers, apparently regardless of the sex ratio (Table 2). This trait of bees (Menzel 1985) and Syrphidae flies (Lunau 1988) associated with the flower’s constancy (Waddington 1983) and learning through visual and chemical stimuli could be correlated with these insects’ avoidance behaviour towards rewardless flowers. The posterior *κ* means were higher than the adopted prior mean (i.e. *μ*_*κ*_ = 10.0) in all experiments (24.5 in the CT treatment, 17.7 in the MFT treatment and 13.3 in the MMT treatment). This could indicate that insects’ accuracies were more similar to each other than to the assumed prior in each treatment. In other words, the insect’s choices were almost identical; such lack of variation between insects suggests that the visiting female flowers’ biases *θ* are strongly dependent on the discriminatory ability bias *μ*.

Data in the present study were structured by insect, and different insects were assigned to a particular condition, visited a particular number of flowers and made the correct choice in a particular number of those flowers. This could mean that insect accuracy differed between treatments; again, the posterior *k*_*e*_ mean values were higher than the assumed prior, thus the individual estimates were also affected by shrinkage, which pulled the individual estimates towards the group’s mean. The shrinkage, therefore, also increased the certainty of the estimated mean of the group (Kruschke 2011).

The DIC values between the herein tested models suggest that the hierarchical naïve model A2-C2 presenting open-minded priors was the best model. In other words, data from one treatment had no influence on data from another treatment, and the parameters of each insect were estimated separately from the estimates of other insects. Conceptually, this means that the random spreading of individual accuracies around the group’s mean accuracy was different in each treatment, although many insects generated similar data proportions. Therefore, the category structure influenced the mean accuracy in the treatment, as well as the random variation of insects, which was close to the mean accuracy (according to Kruschke’s (2011) proposals).

The assessment applied to differences in DIC value between model B-C2 and the smallest DIC (i.e. model A2-C2) (ΔDIC = 6) provides support for model A2-C2, but this does not mean that this model is good in an absolute sense (McCarthy 2007). The standard deviation *σ*_*γ*_ of the *κ*_*e*_ value distribution was not close to 10.0 (*σ*_*γ*_ = 11.86 [0.55; 28.35]) if one considers model B-C2 as the best model instead of model A2-C2. According to Kruschke (2011), the mean *μ*_*γ*_ of 10.0 and the standard deviation *σ*_*γ*_ of 10.0 allowed reasonable variations between insects’ discriminatory abilities. However, such a variation was not the same narrowness of the *k*_e_ distribution values captured by the estimated standard deviation *σ*_*γ*_. In other words, the treatments presented slightly different within-group variations, and this is the exact outcome suggested by model A2-C2.

Standard deviations close to 10.0 were credible in the posterior, since the three treatments presented apparently different within-group variations; however, the variations were as great as those specified in the current study, according to model B-C2. Such an outcome was attributed to the fact that different insects were assigned to different treatments. However, although pollinators belonging to different species may differ significantly in their discriminatory abilities (Le Corff *et al.* 1998), the present findings indicated that different pollinator species exhibited similar behaviour in each treatment, as well as clearly distinguishing female (mimic flowers) from male flowers (model flowers). Visual pollen and stamen signs, such as UV absorption or yellow patterns, could help this (Lunau 1992, 1993); although white flowers are more easily detected by bees (Lunau *et al.* 2011), small contrasting differences in the background could influence bees’ choices (Bergamo *et al.* 2015). Arnold *et al.* (2010) found variation in UV reflectance in distinct *Begonia* flower parts, and this could be associated with the discrimination of rewardless flowers. Other studies involving *Begonia* spp. have found that male- and female-phase inflorescences show strong visual resemblance, and that pollinators respond to the differences in rewards because they show a remarkable preference for male flowers (Agren and Schemske 1991; Wyatt and Sazima 2011). Although the colour and size of the *B. cucullata* flowers were not measured, future studies could explore this topic in order to help better understand pollinator’s foraging procedures in *Begonia* and other mimetic systems.

### Differences in sex ratio: is there an effect on reproductive parameters?

The sex ratio in natural populations can be determined by different forces such as gamete competition and other reproductive parameters, as well as by environmental quality (Freeman *et al.* 1981) or by physiological traits (Heslop-Harrison 1972). However, the sex ratio in self-mimicry pollination systems may be more difficult to understand in light of classical ecological or physiological factors, because it is associated with the relative abundance of models and mimics in the population. Accordingly, although these factors could be working in synergy, the sex ratio found in the *B. cucullata* population (1:1) may result from the trade-off between sex maintenance costs and pollination quality. Although a low pollinator frequency was found in all three treatments, the expectation to have more visits in male-biased array was partially reached when the fruit set observed in MMT was higher than that in other treatments. However, these results contrast with the higher fruit quality found in CT that must be the result of a higher quantity and quality of legitimate pollen on the stigmas. Such divergence is not consistent with the current prediction of higher fruit set and quality in the MMT treatment, and with a larger number of more male (model) flowers, as found in Castillo *et al.* (2002). The low fruit quality in both biased treatments could result from the extremely low probability of recording visits to female flowers in MMT, and from the small number of pollen donors (male flowers) in MFT (Le Corff *et al.* 1998). In addition, the time spent on each flower and other components of the insect’s behaviour could be associated with differences in fruit quality, although this variable was not observed.

Matthews (1977) highlighted the importance of visitor frequency in the effectiveness of mimicry systems. This factor could be associated with the selective advantage of equal sex ratios (CT) in the *B. cucullata* population at the study site. The sessile and aggregated conditions found in plant populations promote the evolution of mimicry systems (Williamson 1982). Furthermore, a fundamental assumption is that the mimicry system must have more models than the mimic flowers. The promotion of reproductive advantages in this system (Johnson 2000) was not shown by the current results. López and Domínguez (2003) showed a strong association between pollination intensity and sex ratio in *B. gracilis*; thus, the low pollination intensity resulted in a male-biased sex ratio, whereas a high intensity would lead to a female-biased ratio. The present results were not consistent with their findings, because *B. cucullata* presented a high pollination intensity, absence of PL and an unbiased sex ratio in the natural population.

## Conclusions

The current findings show the reproductive advantages of an even model mimic and sex ratios in plant populations, as well as in other similar deceit systems (Agren *et al.* 1986). Female flowers are less abundant than male flowers in most self-mimicry systems where only male flowers are rewarding; thus, there are many more models than mimics. We did not observe greater pollination efficiency in female-biased populations. The Bayesian approach allowed us to infer accurately the low female-visit frequency in all treatments. Avoidance of rewardless female flowers by honeybees (the most frequent floral visitor in all sex ratio arrays) reinforces the particular self-mimicry traits in *B. cucullata*, and highlights the importance of other floral cues that signal the true reward source in this plant species. Thus, the high fruit set observed in the *B. cucullata* population may result from other ecological/biological factors, rather than from the efficacy of the self-mimicry pollination mode. Possible factors include the number of pollen grains reaching the stigma surface in relation to the number of ovules, the amount of pollen deposited during single visits, the effects of the even floral sex ratio in the study species and the extreme generalist foraging by pollinating honeybees.

## Sources of Funding

We did not receive funding for this research.

## Contributions by the Authors

R.S.A. designed the study; R.S.A., S.S.O., B.M. performed the study; R.S.A., S.S.O., J.R.I.R. analysed data; and R.S.A., S.S.O., B.M., J.R.I.R. wrote the paper.

## Conflicts of Interest

None declared.

## Supporting Information

The following additional information is available in the online version of this article—

**Table S1.** Total number of visits to female and male *Begonia cucullata* flowers per treatment.

**Figure S1.** Scatterplots showing posterior samples of *μ* and *κ* in the three differential sex ratio experiments from JAGS (Just Another Gibbs Sampler), in the hierarchical naïve model A2 (C2) with open-minded priors [i.e. *μ* ~ *β*(*μ* | 1, 1)]. 2A, the ‘control treatment’ (CT); 2B, the male-biased experimental population (MMT); 2C, the female-biased experimental population (MFT).

## Supplementary Material

Supporting-Information
