# Supplementary material for: Effects of model-mimic frequency on insect visitation and plant reproduction in a self-mimicry pollination system
Source: AoB Plants. 2017 Oct 11;9(6):plx044. doi: 10.1093/aobpla/plx044 (PMC5724025; doi:10.1093/aobpla/plx044)
Supplement: Supporting-Information [file plx044_suppl_supporting-information.docx]

| **Table S1.** Total number of visits in female and male flowers per treatment in *B. cucullata*. | | |
| --- | --- | --- |
| Treatment | Total number of visits | |
|  | Male | Female |
| CT | 384 | 43 |
| MFT | 154 | 28 |
| MMT | 175 | 12 |

**
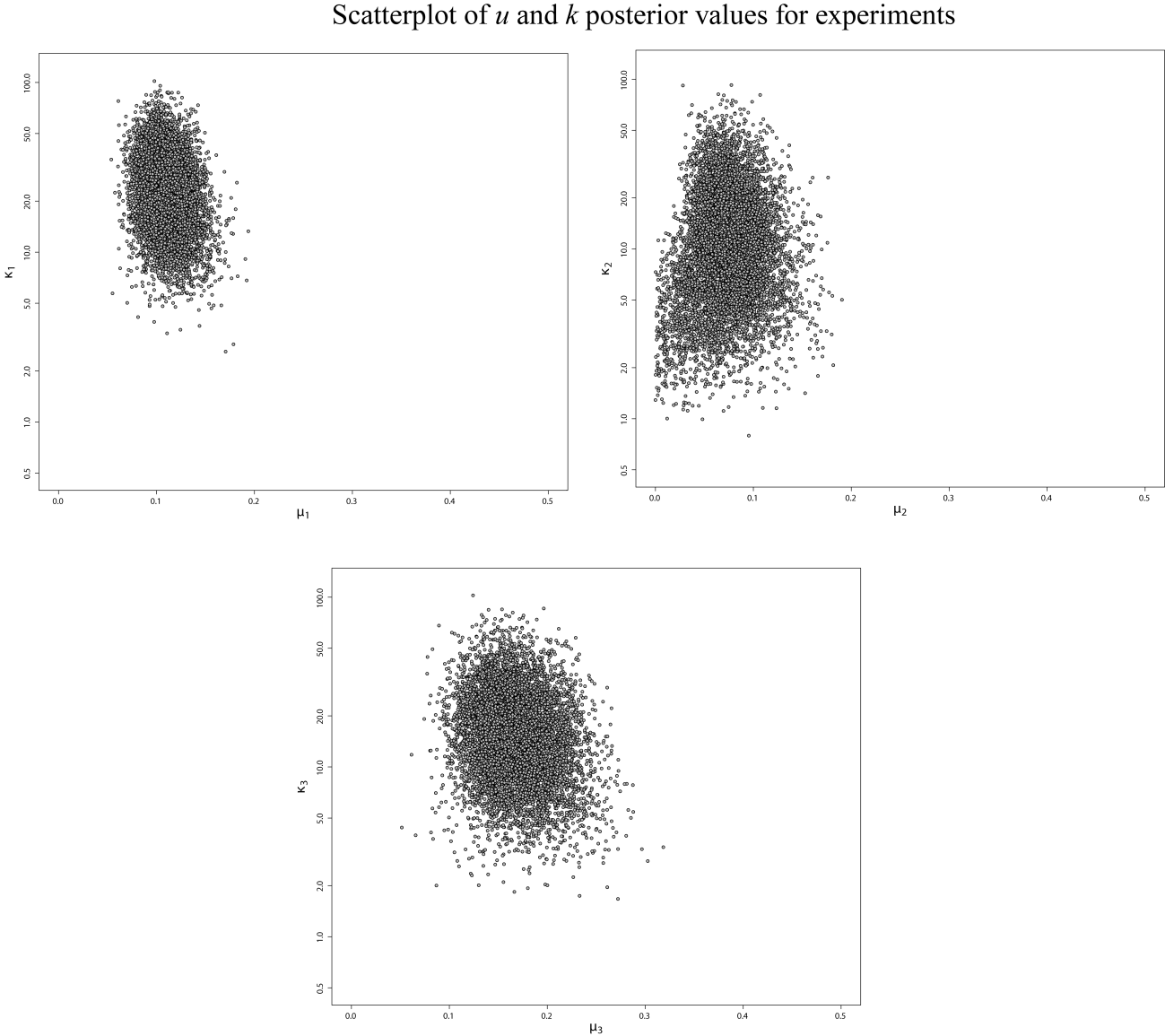
**

**Figure S1.** Scatterplots showing posterior samples of *μ* and *κ*in the three differential sex ratio experiments from JAGS (Just Another Gibbs Sampler), in the hierarchical naïve Model A2 (C2) with open-minded priors [i.e., *μ*~beta(*μ*|1,1)]. 2A, the “control treatment” (CT); 2B, the male-biased experimental population (MMT); 2C, the female-biased experimental population (MFT).
